# Satisfaction with Delivery Services Offered under the Free Maternal Healthcare Policy in Kenyan Public Health Facilities

**DOI:** 10.1155/2018/4902864

**Published:** 2018-05-22

**Authors:** C. M. Gitobu, P. B. Gichangi, W. O. Mwanda

**Affiliations:** University of Nairobi, Kenya

## Abstract

**Background:**

Patients' satisfaction is an individual's positive assessment regarding a distinct dimension of healthcare and the perception about the quality of services offered in that health facility. Patients who are not satisfied with healthcare services in a certain health facility will bypass the facility and are unlikely to seek treatment in that facility.

**Objective:**

To determine satisfaction level of mothers with the free maternal services in selected Kenyan public health facilities after the implementation of the free maternal healthcare policy.

**Methods:**

Data was collected through a quantitative exit survey questionnaire. The respondents were mothers who had delivered in the health facilities and were waiting to leave the health facilities after discharge. The sample included 2,216 mothers in 77 public health facilities across 14 counties in Kenya under tier 3 and tier 4 categories. The number of respondents to be interviewed was proportionately arrived at based on each health facility's bed capacity.

**Results:**

The study established a satisfaction rate of 54.5% among the beneficiaries of the free maternal healthcare services in the country. Mothers benefiting from the free delivery services were satisfied with communication by the healthcare workers, staff availability in the delivery rooms, availability of staff in the wards, and drug and supplies availability (>56%) but unsatisfied with consultation time, cleanliness, and privacy in the wards (<56%). High education levels and lengthy stay in healthcare facilities were negatively associated with the satisfaction with the free delivery services (*P* < 0.05).

**Conclusion:**

There is a high satisfaction with the free maternal healthcare services in Kenya. However, the implementation of the free maternal healthcare policy was associated with low privacy, poor hygiene, and low consultation time in the health facilities. Therefore there is need to address these service gaps so as to attract more mothers to deliver in public health facilities.

## 1. Background

Millennium Development Goal (MDG) 5-Target 5A called for the reduction of maternal mortality ratio by three-quarters between 1990 and 2015; however, to date, reduction of avoidable pregnancy related mortalities still remains one of the greatest health challenges and priorities globally [1]. In the year 2015, there were 303,000 maternal mortalities worldwide [1]. Despite numerous global efforts to curb these pregnancy related deaths, they remain disturbingly high in developing countries yet they are almost always preventable through the attendance of pregnancy and deliveries by skilled healthcare professionals in adequately supplied and equipped health facilities [2].

Maternal mortality has been shown to occur as a result of direct obstetric causes (73%) and indirect causes (27%) with the major causes being haemorrhage (27.1%), hypertensive disorders (14%), sepsis (10.7%), abortion (7.9%), and embolism (3.2%) with these figures showing regional variations [3]. Thus, the Global Strategy for Women's, Children's and Adolescents' Health (2016–2030) envisions a world in which every woman, child, and adolescent in every setting realizes his/her rights to physical and mental health and well-being, has social and economic opportunities, and is able to participate fully in shaping prosperous and sustainable societies [4].

Kenya is still struggling to reduce maternal mortality rate in the country which stands at 362 deaths for every 100,000 live births [5]. This high maternal mortality rate has been partially attributed to unskilled deliveries in the country (38%) which are conducted outside health facilities [5]. Low utilization of maternal healthcare services in Sub-Saharan Africa has for long been linked to the high cost of the services [6, 7]. In Kenya, cost of maternal and child health services has also been cited as one of the obstacles to service utilization [8, 9].

Unaffordable health services globally are a result of inadequate health financing care models which often result in catastrophic expenditures throughout pocket payments for health services [10–16]. At the 13th of December 2017, at least half of the world's population could not access essential health services due to cost implications while 800 million people globally spend at least 10 percent of their household budgets on health expenses for family members [17]. For this reason, universal health coverage has been suggested as an additional measure to promote access to health services by all individuals and communities without suffering financial hardships and it entails the full spectrum of essential, quality health services, from health promotion to prevention, treatment, rehabilitation, and palliative care [18, 19].

Economic related reasons are a significant barrier to delivering at health facilities in many regions in Kenya [15, 16]. Previous assessments in Kenya have established that up to 11% of women do not deliver in health facilities due to the cost of skilled delivery services [20]. Catastrophic spending on health is approximated to be 6.2% in Kenya given that only 17.1% of Kenyan households had medical insurance covers in 2013 [21] and 20.6% had medical insurance covers in 2014 [22]. The Kenya household expenditure survey 2013 reported that wealthy individuals in the richest wealth quintile were more likely to seek heathcare services when compared to poorer individuals in low wealth quintiles [21]. This report further shows that the richest wealth quintile were more likely to utilize inpatient care specifically, 56 admissions per 1,000 population against 28 admissions per 1,000 population for the poorest quartile.

Based on the observations of economic barriers to health services utilization observations, there have been proposals and resolutions to streamline health financing systems to promote access to quality health services [23]. In addition, the 2006 healthcare financing strategy for the African countries by WHO emphasizes the expanded coverage of healthcare services to ensure accessibility by all African populations [24]. As a result of this, African countries such as Mali, Ghana, Uganda, South Africa, Niger, Senegal, and Burkina Faso have been undertaking healthcare financing reforms such as abolishment of user fees in health to improve the accessibility of healthcare services [25].

It is against this backdrop that the Kenyan government introduced a free maternal healthcare policy through a presidential directive on 1st June, 2013, so as to promote skilled delivery and reduce pregnancy related mortalities [26]. Through this policy intervention, mothers are supposed to deliver free of charge in Kenyan public health facilities. Public health facilities are reimbursed between 25 and 175 US dollars for every delivery whether through spontaneous vaginal delivery or caesarian section. The amount of money reimbursed to the health facilities for every delivery is based on the facility status and health systems capacity to handle complications. As such, tier 1 health facilities, where no caesarian sections, blood transfusion, or inward referral of complicated cases are carried out, receive the minimum amount for every delivery (25 US dollars). Tier 4 health facilities handle most complications from deliveries and therefore they receive the maximum 175 US dollars. It is expected that mothers will be encouraged to deliver in health facilities, maintain a good relationship with the health facilities, and continue to seek other services such as postnatal care services thus ultimately improving pregnancy outcomes. Initial assessments reveal that the free maternal health policy implementation in Kenya is faced with numerous challenges among them shortage of drugs and supplies, insufficient funding, shortage of skilled healthcare workers, noninvolvement of stakeholders in maternal health, late reimbursement of the costs incurred in providing free maternal healthcare services, heavy workloads, and demotivation of health workers [27, 28].

The existence of free delivery healthcare service neither promises their utilization by women nor does it guarantee optimal pregnancy outcomes or satisfaction with the services [29]. Reproductive health services should be satisfactory to mothers since they may have immediate and long-term effects on her health, subsequent utilization of delivery services, and recommendation of the services to her peers [30–32]. It is for this reason that the World Health Organization (WHO) recommends a close monitoring of women's satisfaction with delivery services as a quality check to improve skilled delivery outcomes [33]. Patients' satisfaction with healthcare services is one of the measures for quality of care that has been shown to influence confidence in a health facility and the subsequent utilization of services from the facility [34, 35]. Patients' satisfaction with quality of healthcare is the degree to which the patients' desired expectations, goals, and preferences are provided by the healthcare service providers [36]. Patients' satisfaction and dissatisfaction with healthcare services indicate their perception about the strengths and weaknesses in the service delivery [37].

Satisfaction with healthcare services in Kenya has for long been reported as low and cost of services has been given as one of the reasons for dissatisfaction with the services [32, 38–40]. Similarly, incidences of unfriendly healthcare workers as well as poor treatment of pregnant mothers in public health facilities in Kenya have been demonstrated to be a barrier in maternal healthcare seeking behaviour [27, 28, 41, 42]. This study was intended to establish the satisfaction of beneficiaries with free delivery services in Kenyan public health facilities and identify factors associated with the satisfaction. Recall questions were used to establish the satisfaction with delivery services by mothers who had their previous deliveries in the public health facilities prior to the policy intervention.

## 2. Methods

### 2.1. Study Design

This study employed a cross‐sectional analytical approach. A structured questionnaire was used to interview mothers through exit interviews in the public health facilities between July and December 2015.

### 2.2. Dependent Measures

Satisfaction in this study was based on the following variables: consultation time, communication, and attitude by the healthcare workers, staff availability in the delivery rooms, availability of staff in the wards, cleanliness in the health facilities, drug and supplies availability, and privacy in the wards. These variables were selected based on previous studies in the country which revealed that they were the major aspects of delivery services that influenced user satisfaction [14, 38, 40].

Beneficiaries were asked to score each satisfaction element on a Likert scale of 1 to 5 (where 1 was very low, 2 was low, 3 was moderate, 4 was high, and 5 very high). The threshold for satisfaction was set as 56% of the respondents for each dependent measure given that a previous study on satisfaction in the county had established delivery service user satisfaction to be 44% with 12% of the respondents being unsatisfied with the cost of services charged [32].

### 2.3. Study Population

The respondents were mothers who had delivered in the health facilities, had been discharged from the health facilities, and were waiting go home.

### 2.4. Study Setting

Kenya is administratively divided into 47 counties [27]. The public health facilities in the country are categorized into four tiers; tier 1 is community health centres, tier 2 is primary care level health facilities, tier 3 is county and subcounty health facilities, and tier 4 is the national referral health facilities [43].

### 2.5. Selection of Health Facilities

Stratified multistage sampling was used to select 77 public health facilities in 14 of the 47 counties in Kenya (5 high risk, 5 medium risk, and 4 low risk maternal mortality risk counties based on annual maternal mortality figures for the years 2008, 2009, and 2010).

Fourteen of the forty-seven counties in the Republic of Kenya were selected for inclusion in the study after single-stage cluster sampling and subsequent simple random sampling procedures were applied [44].

The 47 counties were classified into high risk, medium risk, and low risk maternal mortality categories based on their perennial maternal mortality ratios. Of these counties, five with a high risk (maternal death to females population ratio above 0.00018), five with a medium risk (maternal death to females population ratio between 0.00012 and 0.000183), and four with a low of risk maternal mortality (maternal death to females population ratio below 0.00012) were included in the study; these studies were selected via simple random sampling. As at 2015, Kenya had a total of 234 health facilities in tiers 3 and 4 of which 97 were in the 14 selected counties, from which 77 (76 in tier 3 and 1 in tier 4) were selected (based on 5% margin error, 95% confidence interval and a postulated increase in satisfaction from 54% to 56% following elimination of delivery charges) through stratified multistage sampling with the maternal mortality risk, counties, status of health facilities and location being the strata (whether urban or rural as classified in the master health facility list), and the proportion of health facilities in each county [45, 46].

### 2.6. Selection of Respondents

2,217 respondents were proportionately divided among the health facilities based on the bed capacity in each health facility (based on 5% margin error, 95% confidence interval and a postulated 56% satisfaction rate). Systematic random sampling of respondents was carried out with the *n*th respondent in every health facility being the number of free maternal healthcare services beneficiaries eligible for inclusion into the study divided by the sample size allocated for the facility. Where a health facility had more than one maternity ward, the total population of respondents was sum number of eligible respondents in all the maternity wards.

### 2.7. Exclusion Criteria

Mothers who declined to participate were excluded from the study and so were mothers who had not delivered and those who had not been discharged at the time of the interviews. The respondents for the exit survey were mothers who had delivered in the selected public health facilities, were discharged, and were waiting to go home (after utilizing the free maternal healthcare services); only those who consented to participate in the exit survey were included in the study.

### 2.8. Recruitment Strategy

Nurses in charge of maternity wards were used to identify and recruit respondents. All the background information regarding the study (including benefits and risks) was provided to each mother; they were requested to give consent for participation in the study.

### 2.9. Instruments of Data Collection

The data collection tool used in this study was an interviewer who administered questionnaire for the beneficiaries of the free skilled delivery services. The questionnaire ranked the responses to satisfaction questions through a Likert scale.

### 2.10. Data Management and Statistical Analysis

Four research assistants collected the data after rigorous training on data collection and research ethics by the main author using the National Institutes of Health (NIH) research ethics guidelines [47]. Pretesting of the data collection tools and pilot testing of the data collection procedures were done at a level 4 health facility outside the sampling frame. Data analysis was carried out using the Statistical Package for Social Sciences (SPSS) version 23 and graphics were generated using Microsoft Excel (version 2013) while the degree of association between responses was tested at 95% confidence interval through Pearson's Chi-square test. Data was collected in Kiswahili and English languages in the months of June to October 2015.

### 2.11. Ethical Considerations

Ethical approval was obtained from Kenyatta National Hospital and University of Nairobi Ethical Committee while administrative approval was obtained from the Ministry of Health headquarters in Kenya, county health officials, and the health facility administrators. Those who consented to participate in the study were interviewed in isolated rooms to ensure privacy. The study participation was voluntary; the nursing officer in every ward acted as witness to ensure that only those who gave consent were included in the study. The participants signed a consent form upon satisfactorily understanding the scope of the study and consented to participate in the study; where mothers below 18 years were encountered, they gave assent and their parents signed the consent forms. There was no direct or indirect compensation for participation in the study.

## 3. Results

### 3.1. Response Rate

Out of the 2,217 questionnaires administered, 2,216 were completed translating to a 99.6% response rate. Of these, 82.2% of the respondents were interviewed in tier 3 health facilities while 17.8% were interviewed in tier 4 health facility. In addition, 57.7% of the health facilities were located in urban areas while 42.3% of the health facilities were located in the rural areas.

### 3.2. Demographic Information of the Respondents

The study population had a median age of 26 years, a minimum age of 13 years, and a maximum age was 49 years (Table 1). Among these respondents, 72.7% were married females, 24.1% single females, 2% divorced, and 1.2% widowed. In addition, 89% of the respondents were Christians while 9.7% were Muslims, 0.5% Hindus, 0.4% subscribed to traditional believes, and 0.4% belonged to other unspecified religions. In terms of level of education levels, 27.2% of the mothers had attained a tertiary level of education, 24.3% of the mothers had completed secondary school education, and 3.2% of the respondents had never been to school.

### 3.3. Satisfaction with Free Maternal Healthcare Services

**Figure 1:**
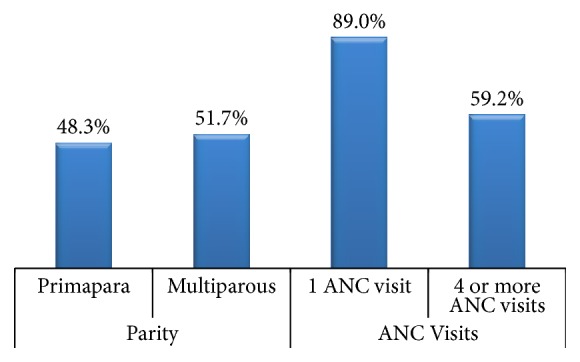
Clinical characteristics of respondents.

On analysis of previous delivery histories, 48.3% were primipara (first time mothers) while 51.7% were multiparous respondents. At least 1 antenatal clinic (ANC) visit was reported by 89.0% of the mothers. In addition, 59.2% of the mothers had made 4 or more ANC visits while 40.8% had made less than 3 ANC visits (Figure 1). Respondents got to know of the free delivery services through the media (37%), healthcare workers (15.7%), relatives (22.4%), mobile outreaches (6.9%) through neighbours (10.7%), friends, workmates and community health volunteers (6.2%), and undisclosed sources (0.5%) while 0.4% were not aware of the existence of free delivery services.

The median duration of stay in the health facilities before discharge was 3 days (3 days for those who had spontaneous vaginal deliveries and 4 days for those who delivered via caesarian sections) while the maximum and minimum days spent in the health facilities before discharge were 70 days (28 days for those who had spontaneous vaginal deliveries and 70 days for those who delivered via caesarian sections) and 2 days (1 day for those who had spontaneous vaginal deliveries and 2 days for those who delivered via caesarian sections).

The study established that 54.5% of the respondents were satisfied with delivery services offered through the free maternal health policy while 30.2% and 15.3% remained neutral and were unsatisfied about the services, respectively. The only service elements that the mothers were not satisfied with (<56%) were consultation time, cleanliness, and privacy in the wards (Table 2).

The satisfaction levels were highest in tier 4 health facility (65.1%) when compared to tier 3 health facilities. Satisfaction with privacy was higher in tier 4 health facility (47.6%) when compared to tier 4 heath facilities (30.8%). There were no differences in satisfaction levels between mothers receiving free maternal healthcare services in rural and urban areas (Table 3).

Sharing of beds by mothers during their stay in health facilities was reported by 43% of the mothers while 26.8% of the mothers reported that their babies shared incubators with other babies in the health facilities. Sharing of beds and incubators was highest in tier 4 health facility at 62% and for incubators at 51.1%, respectively. A large proportion of the respondents (85.7%) reported to have received explanations for procedures carried out on them and information on drugs that were administered or prescribed (85%) with the highest proportion being in tier 4 health facility (94.9% and 93.2%). In addition, 49.3% of the mothers reported to have been provided with a mosquito net while 52.8% were provided with warm water for showering.

A bivariate analysis of factors associated with satisfaction revealed that that level of facility, days spent at the health facility, number of ANC visits, time taken to access a health facility, sharing of beds, sharing of incubators, effectiveness of pain control remedies, explanation of procedures to be carried out, provision of information on drugs, quality of meals, cleanliness of sanitation facilities, provision of warm water, and waiting time before being attended to upon arrival at the health facilities were predictors of satisfaction (*P* < 0.05). From these factors, a lengthy stay in the health facilities (*P* = 0.01) and high levels of education level (*P* = 0.01) were associated with dissatisfaction with the free maternal healthcare services (Table 4).

## 4. Discussion

This study was aimed at establishing users' satisfaction of delivery services offered under the free maternal healthcare policy in Kenyan public health facilities. The minimum length of stay in the health facilities was 2 days with a mean of 3 days and maximum of 70 days spent in the health facilities. This observation is in agreement with findings that 87% of free maternal healthcare beneficiaries had a length of stay of up to two days at the Nakuru provincial hospital while 13% of them left the health facility just a day after admission [48]. The Kenya National Commission on Human Rights noted that some mothers were being discharged earlier than recommended due to overcrowding in maternity wards due to limited beds in view of the high demand for free delivery services in Kenyan public health facilities [49]. Whereas shorter durations of stay in health facilities are recommended as part of service efficiency [50], mothers utilizing the free maternal healthcare services stayed in health facilities for an average of 3 days which is in line with the recommended postpartum hospital stay of 48 hours for uncomplicated vaginal delivery [51].

The study shows that beneficiaries of the free maternal healthcare policy are satisfied with communication by the healthcare workers, staff availability in the delivery rooms, availability of staff in the wards, and drug and supplies availability but unsatisfied with consultation time, cleanliness, and privacy in the wards. Low consultation time and poor hygienic environment may be attributed to a high demand for free delivery services against the few available healthcare workers while low privacy in health facilities could have been a result of sharing beds and incubators reported by the survey respondents [27]. A best fit linear regression model for the regressed variables is satisfaction = 12.674 − 0.108 (days spent in the health facilities) + 0.260 (number of ANC visits) + 0.850 (sharing of incubators by newborns) + 0.276 (effectiveness of pain control remedies) + 0.399 (quality of meals provided) + 0.845 (waiting time before being attended to) − 1.44 (education level) + Error.

A 54.5% satisfaction rate is in line with a previous study on satisfaction with delivery services which established that 44% of the respondents were satisfied with the delivery services; and of those dissatisfied with the services, 12.1% of the respondents were dissatisfied with the cost implications [42]. This is an indication that cost is a minor factor in predicting satisfaction with delivery services and that there are other factors which account for contributing to satisfaction of users and they have not yet been addressed by the policy. The findings are also in line with a study at the Kakamega provincial hospital in western Kenya that shows that beneficiaries of the free maternal healthcare policy in the hospital were not satisfied with staff attitude (negligence and use of vulgar language), health facility environment, and privacy [52]. Abolishment of user fees in middle and low-income countries increases service utilization and consequently results in low quality of the services offered [53–55]. Low consultation time may be attributed to demotivation of healthcare workers which has been observed in Uganda and Niger following the abolishment of user fees for delivery services [55–57].

There were no differences in satisfaction levels in rural and urban based facilities; however, satisfaction levels were highest in level 6 health facility (65.1%) which is well staffed and equipped [58]. Through other studies, high education levels were negatively associated with satisfaction, a finding that has been reported by beneficiaries of free maternal healthcare services in Kakamega provincial hospital which was included in this study [52]. Waiting time appears to be a key determinant of satisfaction with delivery services, a finding which is consistent with previous studies in Pumwani maternity home, Nyeri and Thika regions of Kenya, two of which were included in the study [38, 40]. Similarly, effectiveness of pain control remedies and provision of adequate information in the health facilities are important in ensuring satisfaction with delivery services just as documented in Ethiopia and Botswana [59, 60].

Other experiences reported by beneficiaries of the free maternal healthcare policy include provision of a mosquito net (49.3%), provision of warm water for showering (52.8%), sharing of beds by mothers (43%), and sharing of incubators by newborns (26.8%) which point at low infrastructure and resource availability in the health facilities implementing the policy, a finding that has been reported by beneficiaries of the free maternal healthcare services at Machakos Level 5 hospital [61]. Following the abolishment of delivery fees in Kenya, beneficiaries reported a 13% dissatisfaction with the availability of beds at the Nakuru provincial hospital [48]. The Kenya National Commission on Human Rights (2013) noted that, due to the increased number of mothers delivering in health facilities following the implementation of the free maternal healthcare policy, maternity wards were overcrowded and some beneficiaries were forced to leave the hospital early to make room for others or even sleep on the floor due to lack of beds [62]. The counties highlighted by previous studies (Machakos and Nakuru) were not included in this particular study.

## 5. Limitations

Client satisfaction surveys are influenced by cultural response bias which is created by social and cultural factors that influence the way people perceive and respond to survey questions [63]. As such the satisfaction levels reported by the beneficiaries of the free maternal healthcare policy are based not only on their experiences in health facilities but also on social cultural factors. To ensure completeness in the responses, the beneficiary exit interviews questionnaires were interviewer administered. We cannot rule out a possible influence of this approach on responses given by the participants. Other limitations include the lack of a policy guideline outlining the full scope of services that should be offered under the free maternal healthcare policy; absence of variables in the questionnaire to capture place of previous delivery that would have aided in the analysis of satisfaction levels pre- and postpolicy implementation; and lack of absence of variables on ethnic and tribal background of respondents which may also influence satisfaction.

## 6. Conclusion

Mothers benefiting from the free delivery services are satisfied with communication by the healthcare workers, staff availability in the delivery rooms, availability of staff in the wards, and drug and supplies availability but unsatisfied with consultation time, cleanliness, and privacy in the wards. For continued use and recommendation of free delivery services to peers in Kenya, there is need to have changes in these institutional oriented independent predictors of satisfaction by the county governments and the national government.

## Figures and Tables

**Table 1:** Social demographics.

*Age*					

Mean	Standard deviation		Maximum	Minimum	

26.0	6.2		49.0	13.0	

*Marital status*					

Single	Married		Widowed	Divorced	

24.1%	72.7%		1.2%	2.0%	

*Religion*					

Christian	Islam	Hindu	Traditional Believes	Other	

89.0%	9.7%	0.5%	0.4%	0.4%	

*Education level*					

Tertiary level	Secondary school (completed)	Secondary school (incomplete)	Primary school (completed)	Primary school (incomplete)	Never been to school

27.2%	24.3%	17.8%	14.0%	13.4%	3.2%

**Table 2:** Satisfaction with services in the health facilities.

Satisfaction Element	Not Satisfied	Neutral Response	Satisfied
Consultation Time	9.7% (215)	35.6% (791)	54.7% (1210)
Communication by the health care workers	8.9% (173)	29.7% (658)	61.4% (1360)
Availability of staff in the delivery rooms	12.6% (281)	27.1% (600)	60.3% (1335)
Availability of Staff in the wards	14.9% (328)	23.2% (515)	61.9% (1373)
Cleanliness in the health facilities	19.0% (421)	40.7% (903)	40.5% (892)
Privacy in the wards	23.3% (515)	37.1% (825)	39.6% (856)
Availability of drugs and supplies	18.5% (410)	18.1% (402)	63.4% (1404)
Mean for the total	15.3%	30.2%	54.5%

**Table 3:** Analysis of satisfaction against major themes.

Satisfaction Element	Rural based health facilities	Urban based health facilities	Tier 3 health facilities	Tier 4 health facility
Availability of Staff in the delivery room	61.7%	59.2%	59.0%	84.6%
Availability of staff in the ward	61.5%	62.3%	61.2%	80.3%
Availability of drugs and supplies	62.3%	64.1%	67.7%	78.7%
Privacy	39.1%	39.9%	30.8%	47.6%
Cleanliness	39.2%	41%	36.5%	45.6%
Communication	62.1%	60.8%	64.5%	62.8%
Consultation time	54.2%	54.9%	60.3%	56.2%
Overall Satisfaction	54.30%	54.60%	54.3%	65.11%

**Table 4:** Regression output.

Model	Unstandardized Coefficients		Standardized Coefficients	*t*	Sig.	95 % Confidence Interval for *B*	
	*B*	Beta				Lower Bound	Upper Bound
Constant	12.674	0.859		14.755	0.000	10.989	14.359
Days spent at the hospital	−0.108	0.031	−0.087	−3.451	0.001	−0.170	−0.047
Number of ANC visits	0.260	0.054	0.124	4.856	0.00	0.155	0.366
Incubator sharing	0.850	0.250	0.087	3.405	0.001	0.360	1.340
Pain control remedies	0.276	0.108	0.070	2.561	0.011	0.064	0.487
Meals	0.399	0.137	0.088	2.914	0.004	0.130	0.668
Sanitation	1.643	0.136	0.359	12.066	0.00	1.376	1.910
Warm shower water	1.119	0.223	0.133	5.023	0.00	0.682	1.556
Time before attendance	0.845	0.101	0.222	8.375	0.00	0.647	1.043
Education level	−0.144	0.070	−0.053	−2.067	0.039	−.281	−.007

## Data Availability

The complete dataset used in this study is in the custody of Dr. Gitobu Cosmas Mugambi and is available to researchers upon written request.

## List of Abbreviations

ADDRF: African Doctoral Dissertation Research Fellowship
ANC: Antenatal Clinic
BSc: Bachelor of Science
BDS: Bachelor of Dental Surgery
IDRC: International Development Research Centre
MBChB: Bachelor of Medicine, Bachelor of Surgery
MD: Doctor of Medicine
MDG: Millennium development goal
MMED: Master of Medicine
NIH: National Institutes of Health
PhD: Doctor of Philosophy
PMRCPath: Postgraduate Course, Royal College of Pathologists
SPSS: Statistical Package for Social Sciences
UNITID: Institute of Tropical and Infectious Diseases
USD: United States dollar
WHO: World Health Organization.
